# Medio-lateral stability during walking turns in older adults

**DOI:** 10.1371/journal.pone.0198455

**Published:** 2018-06-05

**Authors:** David Conradsson, Caroline Paquette, Erika Franzén

**Affiliations:** 1 Karolinska Institutet, Department of Neurobiology, Care Sciences and Society, Division of Physiotherapy, Stockholm, Sweden; 2 Function Area Occupational Therapy & Physiotherapy, Allied Health Professionals Function, Karolinska University Hospital, Stockholm, Sweden; 3 Department of Kinesiology and Physical Education, McGill University, Montreal, Quebec, Canada; 4 Centre for Interdisciplinary Research in Rehabilitation, Montreal, Quebec, Canada; University of Illinois at Urbana-Champaign, UNITED STATES

## Abstract

**Introduction:**

Medio-lateral stability during walking turns relies on the interaction between precise weight shifts of the body and changes in base of support by regulating step width. Although older adults and clinical populations often slow down while turning in order to compensate for balance impairments, little is known about the influence of walking speed on stability during turning.

**Objective:**

To compare medio-lateral stability between walking turns and straight walking and to investigate whether walking speed affects medio-lateral stability during turning in healthy older adults.

**Methods:**

Nineteen older adults walked straight or walked and turned 180° to the right and left at their comfortable speed and at a slow pace. The walking direction was visually cued before they started to walk (preplanned) or while walking straight (unplanned). As a proxy for medio-lateral stability, we calculated the absolute difference between pelvis lateral displacement and the lateral edge of the base of support during straight walking and turning.

**Results:**

Overall, irrespective of turning condition, medio-lateral stability was enhanced during turning as the pelvis was further away from the boundary of the base of support resulting in a greater margin of stability compared to straight walking. Turning at a slow pace hampered medio-lateral stability as demonstrated by pelvis lateral displacement closer to the boundaries of the base of support resulting in reduced margins of stability. The reduction in stability was caused by a narrower step width during slow walking whereas pelvis lateral displacement was unaffected by turning speed.

**Conclusion:**

In older adults, medio-lateral stability was augmented during turning compared to straight walking, whereas turning at a slow pace hampered medio-lateral stability. These findings provide insights into the postural strategies used by older adults in order to adapt to the postural challenges of turning and straight walking.

## Introduction

Locomotion in everyday life is rarely performed during steady state walking; in fact, up to 50% of the steps executed each day incorporate turning steps [1]. As turning naturally induces instability to the body, it challenges postural control and can cause falls and injuries among older adults [2, 3]. The high prevalence and serious consequences of falls in older adults [4, 5] highlight the importance of detecting age-related mechanisms of fall-related tasks, such as walking turns.

To maintain body stability, the centre of mass (COM) needs to be controlled with respect to the boundaries of the base of support (BOS) [6]. More specifically, a shorter distance between the COM deviation and the boundary of BOS will result in reduced margin of stability which would denote lower ability to deal with perturbations while standing, and thereby increase the risk of instability [7]. Ultimately, moving COM outside the BOS leads to instability and subsequently also falling if stability is not re-established by appropriate postural adjustments (e.g. increasing BOS by taking a widening step). In contrast to quiet stance, COM is often shifted outside the BOS during turning [8]. Therefore, so as to maintain medio-lateral stability while walking and turning, it is necessary to continuously regulate one’s step width with respect to changes in lateral COM displacement [9, 10].

In healthy young adults, compared to straight walking, turning induces a lateral shift of COM outside the BOS which augments with increased walking speed [8]. However, it is unclear whether older adults regulate medio-lateral stability while turning in a similar way as young adults. Specifically, older adults often turn more slowly and with more rigid trunk movements [11], which may affect medio-lateral stability. Older adults have also demonstrated inflexible adaptation to changes in walking speeds [12, 13] and yet, to our knowledge, the speed-dependency of medio-lateral stability during walking turns has not yet been investigated in this population. Furthermore, previous studies have mainly investigated preplanned turns where the walking direction is known in advance. However, real-life situations often require quick and unpredictable turns, with limited time for planning (e.g. turning to circumvent an unexpected obstacle) [14, 15]. Therefore, it is important also to investigate unplanned–reactive–turns incorporating rapid modifications of the walking pattern [14, 16].

In this study, we aimed to 1) examine medio-lateral stability during pre- and unplanned walking turns compared to straight walking, and 2) investigate the effects of walking speed on medio-lateral stability during turning in healthy older adults. Due to greater postural challenges during turning than straight waking, we predicted that turning would have a destabilizing effect on older adults, and thereby cause them to shift their pelvis closer to the margins of BOS. Greater differences in medio-lateral stability between turning and straight walking were also expected for unplanned turns, due to the challenging features of turning [14]. Furthermore, previous findings have shown that medio-lateral control of COM and step width is closely linked to maintaining stability while walking [17, 18]. Therefore, we expected that pelvis displacement and step width amplitude would decrease proportionally with decreased speed, and would thereby not influence medio-lateral stability.

## Materials and methods

### Participants

Nineteen healthy older adults (7 females; age: 72 years, SD 6 years; Body Mass Index: 25.1 SD 2.9) were recruited at senior organizations and recreational centres. Individuals were eligible for inclusion if they were ≥65 years of age, had a Mini Mental State Examination score of >24 and were free from any medical condition or injury affecting their gait or balance. Screening for gait impairments were based on a standardized interview including question about medical history and ongoing gait impairments, as well as an observation of the walking pattern during straight walking and turning. This study was approved by the Regional Board of Ethics in Stockholm, and all participants provided written informed consent prior to their enrolment in the study.

### Experimental protocol

Participants walked at their self-selected comfortable speed along a 9-metre walking lane where the turning position was marked by two poles [19]. The following tasks were performed: walking and turning 180° to the right or to the left, or walking straight. Participants started each trial 4.65 metres from the turning intersection, a distance sufficient to reach a steady-state walking speed before initiating the turn. Two different types of walking turns (preplanned and unplanned) were performed, as described in a previous publication [19]. Briefly, in the preplanned condition, the walking direction was provided before participants started to walk, while in the unplanned condition, the visual cue appeared approximately one step length (0.6m) prior to the intersection point. For both the pre- and unplanned turns, participants were instructed to walk and turn in the direction indicated by the arrow located straight ahead of them at the end of the walkway, without stopping, taking the closest path to the target. Before the start of data collection, practice sessions were conducted so as to familiarize participants with the procedure. The pre- and unplanned turning condition each contained a total of 15 trials per subject (i.e. five trials–in randomized order–for straight walking, right and left turning). The same data collection was repeated after a short break at a slow walking speed (i.e. slower than comfortable walking, as though they were going for a stroll). Prior to data collection, participants performed practice sessions in order to familiarize them to slow walking turns and to find a targeted slow speed. Subsequently, the slow walking speed was assessed with a handheld stopwatch and by counting the number of steps over a distance of two meters prior to the turn during data collection. In cases where participants deviated from the targeted speed, they were instructed to change their walking speed (i.e. to walk faster or slower).

### Equipment

An eight-camera motion analysis system (Elite 2002, version 2.8.4380; BTS, Milano, Italy) was used to record (100 Hz) the position of 9 spherical retro-reflective markers located on the spinous process of the seventh cervical vertebrae (C7) and bilaterally on the head, acromion, posterior superior iliac spine and heel. Three-dimensional trajectories of the markers were reconstructed using a tracking system (Tracklab-BTS, Milan, Italy). Data were processed and filtered (Butterworth low-pass filter: 7-Hz cut-off frequency) using MATLAB software (MATLAB, 7.4.0, MathWorks, Natick, MA, USA).

### Data analysis

The outcome variables extracted for analysis focused on turning performance (i.e. turning speed, turning rotation and step length) and stability (step width, pelvis lateral displacement and medio-lateral stability). We focused our analysis on the first five turning steps, since most participants completed 160° of the turn within those steps. Turning speed (i.e. the cumulative linear displacements of the C7 marker in the horizontal plane divided by stride time) were calculated as the average speed for the five turning strides and turning rotation (i.e. maximum angular pelvis rotation with respect to the laboratory reference) were calculated for the fifth turning step. Step length was calculated in accordance with Huxham et al., (2006) and the average step length for the five turning steps were analyzed [20].

**Fig 1:**
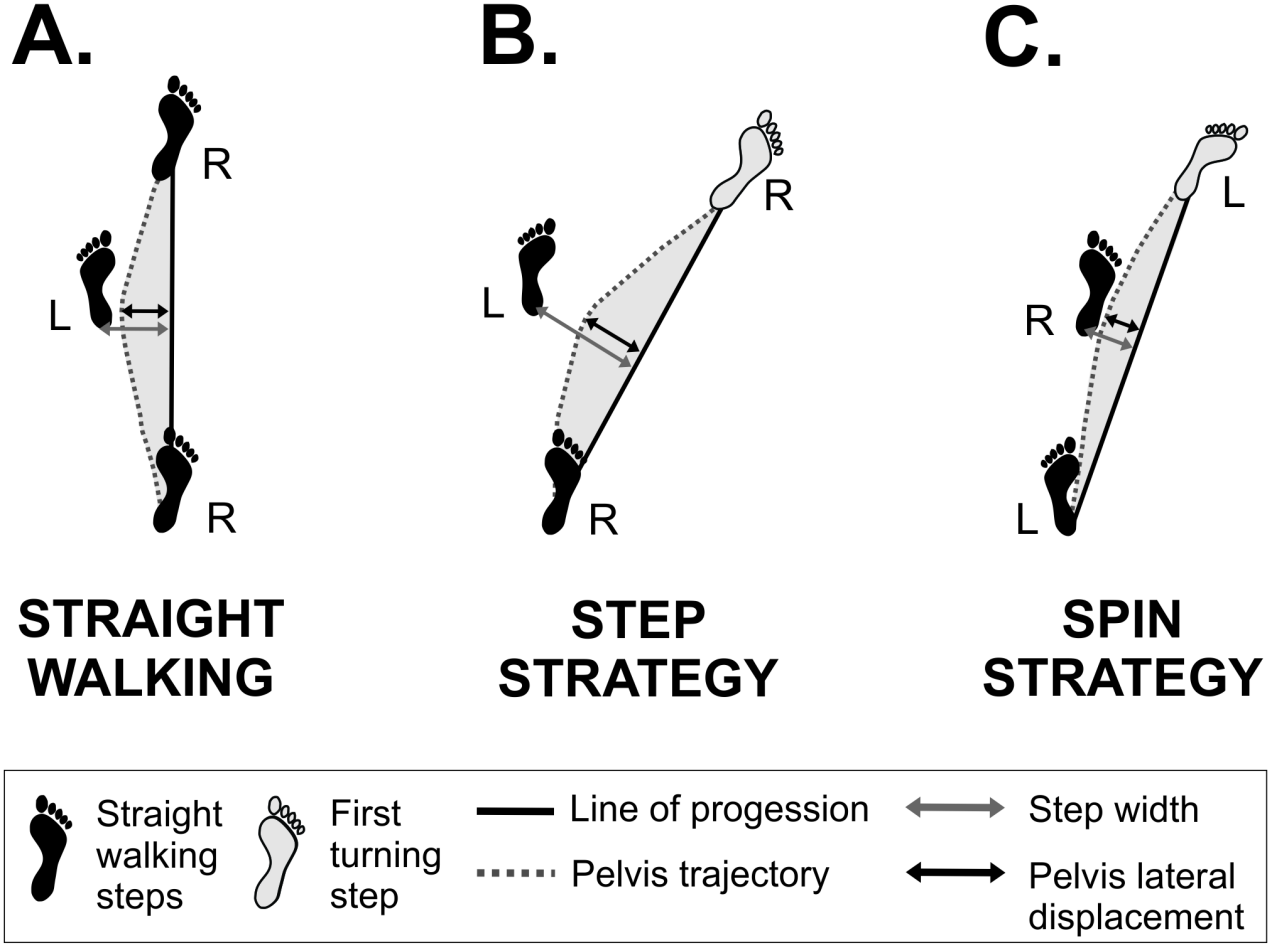
Footstep adjustments during walking and turning. Schematic drawing of representative footstep adjustments for (a) straight walking, (b) step strategy during a right turn (i.e. first turning step with the foot that was on the same side of the body as the turning direction), and (c) spin strategy during a right turn (i.e. first turning step with the foot on opposite to the turning direction). Black foot prints illustrate straight walking steps and grey foot prints show the first turning step. Note that, for the spin strategy, the first turning step (i.e. left/external foot) crossed over and landed medially compared to the progression of the preceding step (i.e. right/internal foot). The solid arrow and the dashed arrow show step width and pelvis lateral displacement with respect to the line of progression of the stride (black line), respectively.

The first turning step was identified as the first heel strike exceeding two standard deviations (SD) in medio-lateral displacement of five straight walking trials (computed for each participant) in the turning direction [19]. Heel strike events were determined based on the velocity profiles of the heel markers (vertical axis). Step width was calculated as the distance between the foot perpendicular with the line of progression (see Fig 1) using the algorithm proposed by Huxham et al., (2006) [20]. To discriminate between preparatory turning steps and the actual step initiating the turn, the succeeding turning step also needed to exceed 2 SD from the straight walking trial average.

We identified two turning strategies: step turns (i.e. first turning step with the foot that was on the same side of the body as the turning direction) and spin turns (i.e. first turning step with the foot opposite to the turning direction) [19, 21]. Similar to straight walking (Fig 1a), a positive step width is used for step turns (Fig 1b), whereas spin turns result in a narrow or negative step width as the external foot (e.g. left foot during a turn to the right) crosses over and lands medial to the line of progression of the internal leg (Fig 1c). As a proxy for COM, the centre of the pelvis segment was calculated as the average position between right and left posterior superior iliac spine [22]. For each gait stride, pelvis lateral displacement was calculated as the maximum distance between the projection of the centre of the pelvis segment and the perpendicular line from the line of progression (see Fig 1a–1c). For straight walking and turning, the maximum distance of pelvis lateral displacement occurred at approximately 80% of the gait cycle, corresponding to midstance [23]. Midstance was used for analysis, since is it is an important phase of the gait cycle for stability, i.e. transfer of COM over the stance limb. As the primary outcome for medio-lateral stability, we calculated the absolute difference between pelvis lateral displacement and step width (positive values: step width > pelvis lateral displacement) for walking and turning strides. This outcome represents the distance between the pelvis and BOS, and larger values reflecting pelvis further away from the boundaries of BOS and closer to the line of progression (see Fig 1a–1c).

### Statistical analysis

Statistical analyses were carried out using IBM SPSS, version 23.0 (SPSS Inc., Chicago, Illinois, USA). For turning performance (i.e. turning speed, turning rotation and step length) and turning stability (medio-lateral stability, step width and pelvis lateral displacement), equality of variance and data normality was tested using Levene’s test, combined with a visual inspection of the normally distributed and residual curve. As no differences were found between turns to the right or left for turning performance or for turning stability these trials were merged for further analysis. For step and spin turns, mixed-model analyses were used to analyze; differences between turning velocity (comfortable vs slow) and turning condition (comfortable vs slow) on average turning speed, rotation and step length. Furthermore, we have previously showed a nearly 50:50 distribution between step and spin turns, and different step width regulation for these turning strategies [19]. Medio-lateral stability, step width and pelvis lateral displacement were, therefore, analysed separately for turning strategies (spin and step turn) and turning conditions (pre- and unplanned). More specifically, a mixed-model analyses was used to analyse the effects of turning velocity (comfortable vs slow) and turning steps (step 1, 2, 3, 4 vs 5) on medio-lateral stability, step width and pelvis lateral displacement. Mean values and 95% confidence intervals (95% CI) were used to compare turning stability between turning and straight. Corrections for multiple statistical testing were not used, due to the criticisms made of such corrections in minimizing the incidence of false negatives when examining hypotheses [24]. Instead, the significance level was set at *P*≤0.025.

## Results

**Fig 2:**
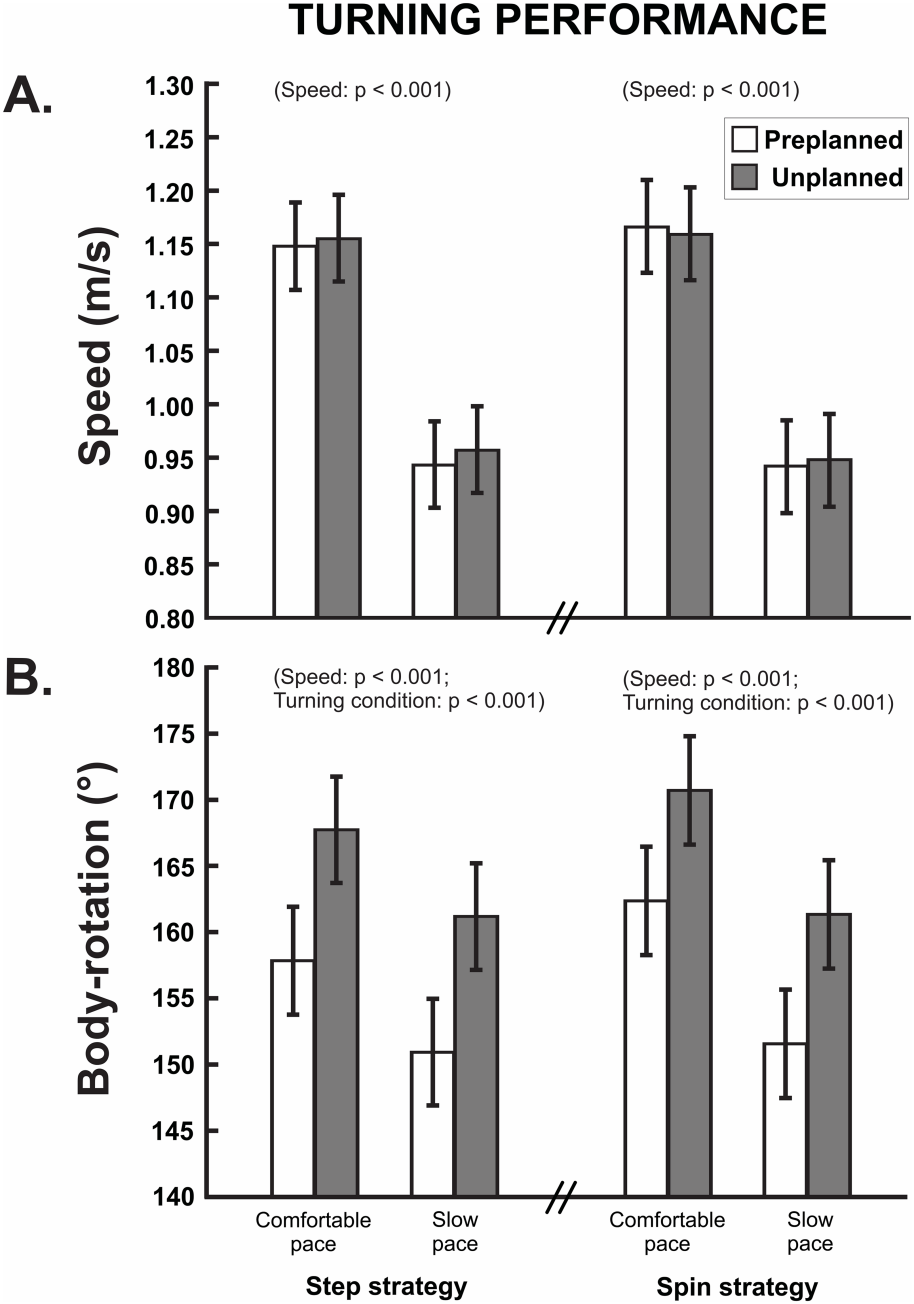
Turning performance. (A) Mean turning speed and (B) maximum turning rotation during pre- and unplanned turning for the comfortable and slow pace condition. Data are presented as mean and 95% confidence interval.

As illustrated in Fig 2a, for step and spin strategy, walking turns at comfortable speed were 21–22% faster during the pre- and unplanned condition compared to turning at slow speed (p < 0.001). Walking turns at comfortable speed also resulted in 4–5% greater rotation for the step strategy (p < 0.001, Fig 2b) and 6–7% greater rotation for the spin strategy compared to turning at slow speed (p < 0.001, Fig 2b). Unplanned turns resulted in 6% higher body rotation than preplanned turns (p < 0.001) whereas there were no differences in turning speed between pre- and unplanned turns (p ≥ 0.382, see Fig 2a). Walking turns at comfortable speed also revealed 12–20% longer average step length for the step strategy (preplanned: 587mm vs 668mm, p < 0.001, unplanned: 560mm vs 670mm, p < 0.001) and the spin strategy (preplanned: 592mm vs 667mm, p < 0.001, unplanned: 599mm vs 670mm, p < 0.001) compared to turning at a slow pace.

**Fig 3:**
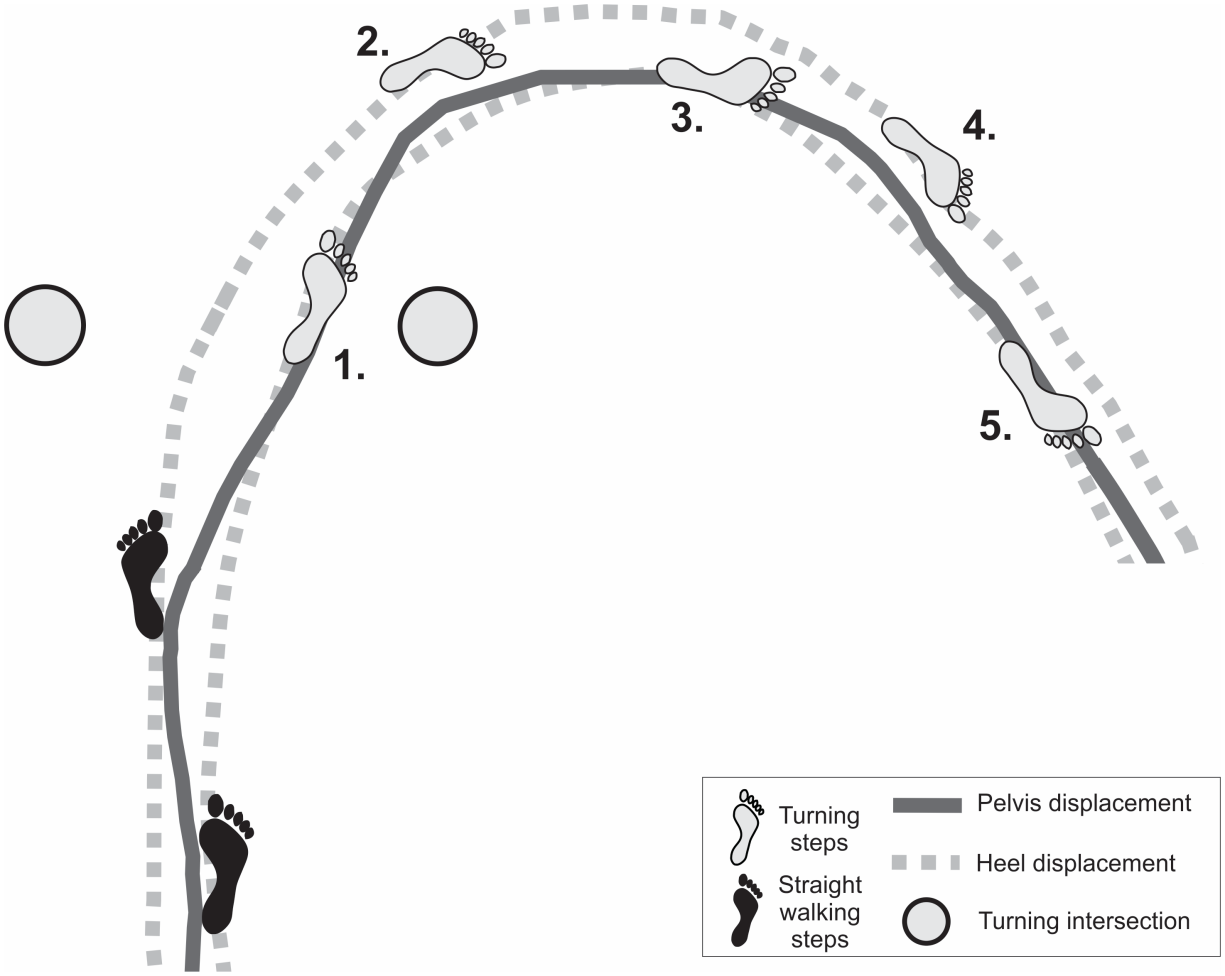
Footstep adjustments and turning trajectory of a right turn. Black foot prints indicate the two pre-turning steps and grey foot prints represent footstep adjustments for five turning steps. Note that similar to straight walking, turning step 1, 3 and 5 reflect widening turning steps with a positive step width, whereas turning step 2 and 4 reflect cross over turning steps where the foot lands close to or medial to the line of progression of the internal leg.

**Fig 4:**
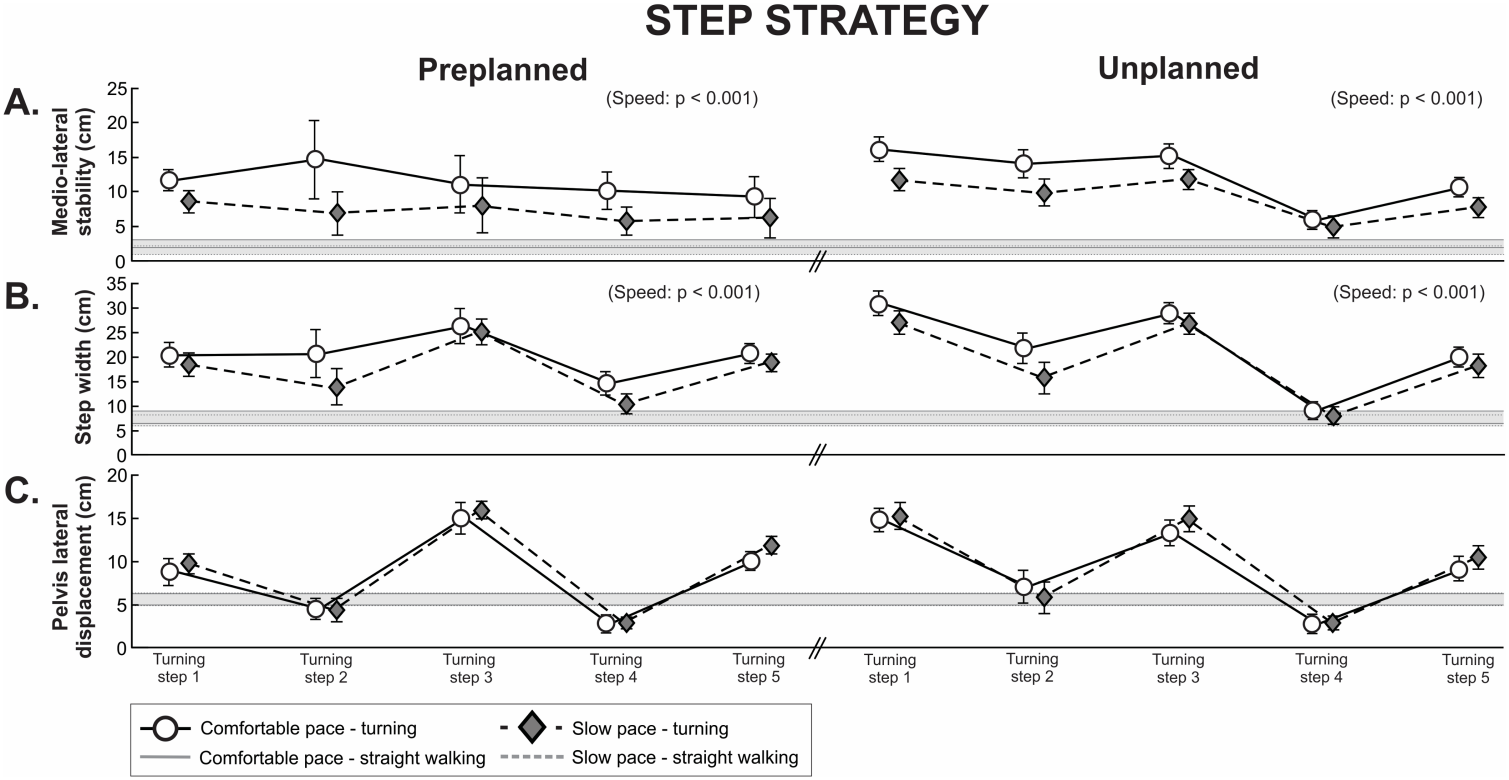
Step strategy. (A) Medio-lateral stability (i.e. the difference between pelvis lateral displacement and step width), (B) step width and (C) pelvis lateral displacement of five turning steps during pre- and unplanned turning for the comfortable and slow pace condition. Data are presented as mean and 95% confidence interval. Grey horizontal solid and dashed lines represent a 95% confidence interval of straight walking for comfortable and slow pace walking, respectively.

Representative turning trajectory and footstep adjustments of right turn is shown in Fig 3. When initiating turning with the step strategy (i.e. widening steps), the distance between the pelvis and the lateral boundaries of BOS was greater for all five turning steps for pre- and unplanned turns than during straight walking (Fig 4a). Turning at a slow pace resulted in pelvis lateral displacement closer to the boundaries of the BOS by 46% for preplanned turns (condition: p < 0.001) and by 22% for unplanned turns (condition: p < 0.001, Fig 4a). This reduction in medio-lateral stability was caused by a significantly narrower step width for preplanned turns (condition: p < 0.001) and unplanned turns (condition: p < 0.001, Fig 4b). Pelvis lateral displacement was unaffected by walking speed for preplanned turns (condition: p = 0.046) and unplanned turns (condition: p = 0.330, Fig 4c).

**Fig 5:**
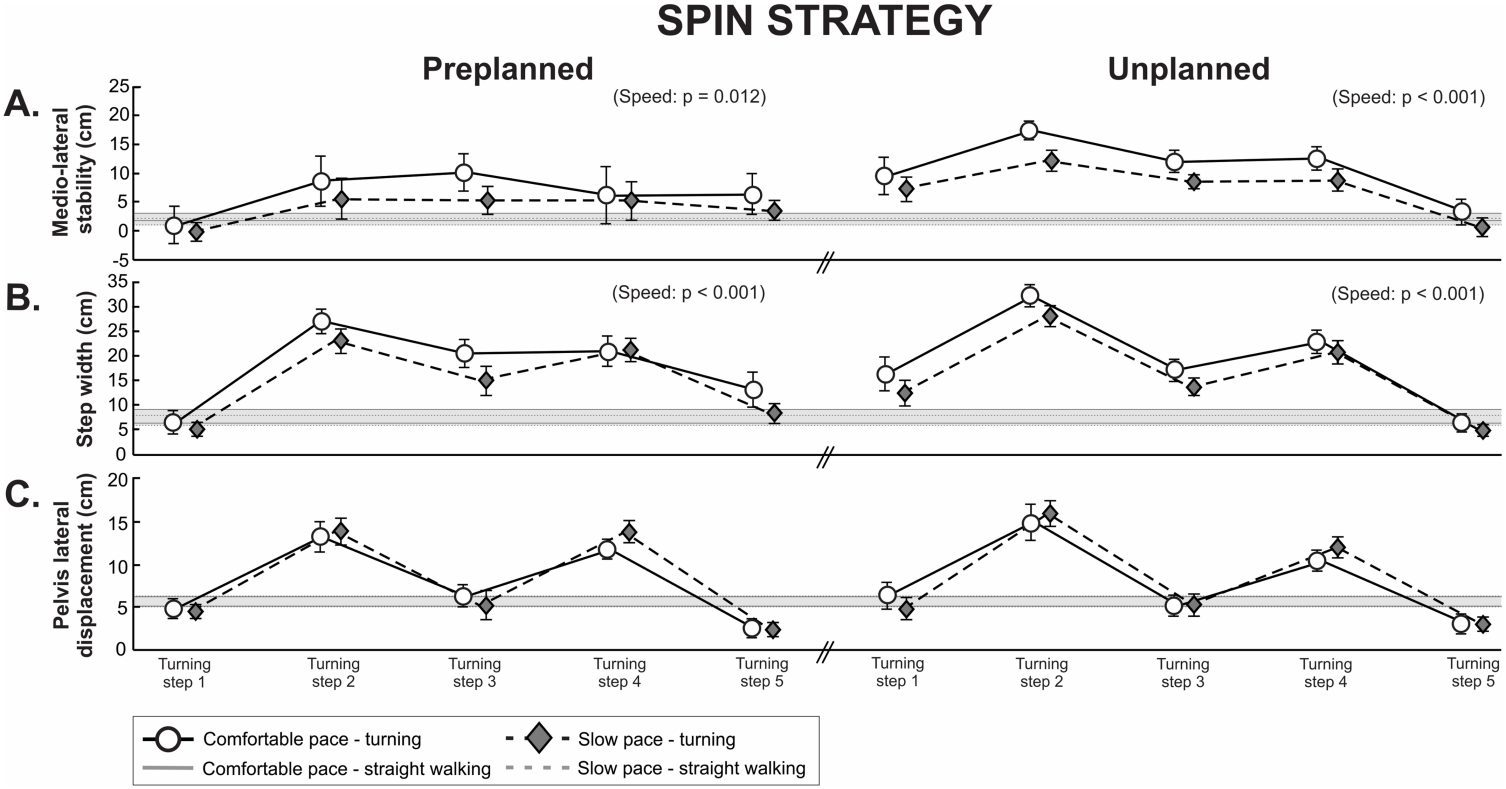
Spin strategy. (A) Medio-lateral stability (i.e. the difference between pelvis lateral displacement and step width), (B) step width and (C) pelvis lateral displacement of five turning steps during pre- and unplanned turning for the comfortable and slow pace condition. Data are presented as mean and 95% confidence interval. Grey horizontal solid and dashed lines represent a 95% confidence interval of straight walking for comfortable and slow pace walking, respectively.

When initiating turning with the spin strategy (i.e. cross-over steps), the distance between the pelvis and the lateral boundaries of BOS was similar to straight walking for most turning steps during the preplanned condition while a greater distance was observed for the first four turning steps during the unplanned condition (Fig 5a). Similar to the results for step turns, initiating turning at slower speed with a spin turn resulted in pelvis lateral displacement closer to the boundaries of the BOS by 51% for preplanned turns (condition: p = 0.012) and by 39% for unplanned turns compared to comfortable walking (condition: p < 0.001, Fig 5a). This reduction in medio-lateral stability resulted solely from a narrower step width for preplanned turns (condition: p = 0.523) and unplanned turns (condition: p = 0.501, Fig 5b).

## Discussion

Contrasting our hypothesis, we found that older adults overall augmented medio-lateral stability while turning by increasing their margins of stability compared to straight walking. On the other hand, turning at a slow pace hampered medio-lateral stability, as the pelvis was shifted closer to or outside the boundaries of BOS, which was caused solely by a narrower step width during slow turning compared to turning at a comfortable speed.

Despite variations in step width and pelvis displacement amplitude, older adults regulated their step width and pelvis displacement to sustain a greater margin of stability during turning than during straight walking. This could reflect an effort to compensate for the greater challenge of maintaining medio-lateral stability during turning. In line with this, compensatory postural strategies are often utilized by older adults, e.g. more rigid control of COM during walking [25] and postural reactions [26], as well as greater step width and increased time in double-stance during straight walking [27, 28].

The spin strategy is usually considered to be more challenging from a postural control perspective due to the narrower BOS and increased demands on axial rotation [29, 30]. However, using the spin strategy during preplanned turns was the only condition where older adults were unable to maintain a larger distance between the pelvis and the margins of BOS during turning than during straight walking (Fig 5a). Owing to the ability to plan and programme movements, preplanned turns have a more proactive nature leading to a smoother transition from straight walking to turning compared to unplanned turns [19]. We speculate that the optimization of movements into more of a steady-state pattern during preplanned turns combined with the narrow BOS of the spin strategy occurred at the expense of medio-lateral stability. However, age-related turning behaviours in relation to the availability of time for motor planning remain to be determined.

We found that older adults reduced medio-lateral stability by using a narrower step width while performing walking turns at a slower pace. As the internal perturbation during turning (e.g. centrifugal force) decreases at lower walking speeds [8], medio-lateral stability that was hampered during slow turns could reflect a strategy adopted by older adults to deal with the lower internal perturbations as compared to their comfortable walking speed. Furthermore, our finding of a narrower step width during slow turning contrasts with previous findings that older adults tend to adopt a greater step width while deviating from their comfortable walking speed during straight walking [31]. Since older adults have been shown to be more vulnerable to instability while walking with a narrow step than young adults [32], it is important to consider regulation of step width in the fall prevention assessment of older adults.

Our findings of the speed dependency of medio-lateral stability during turning are in line with previous findings, which show an effect of turning speed on turning stability [8, 33] and axial coordination [34, 35]. Altogether, these findings emphasize the importance of matching gait speed when comparing turning characteristics between older adults and clinical populations (e.g. neurological diseases) so as to distinguish between changes due to speed and changes due to disease. Furthermore, in contrast to our findings, Orendurrf et al. [8] found that, as turning speed increased, young adults shifted their COM further outside the lateral margins of BOS. These varying findings might be due to age differences or different turning paradigms between the studies. In the current study, turning trajectory and foot placement were not standardized, since we aimed to capture realistic turning behaviour without spatial restrictions. In Orendurrf et al. [8], turning was guided by a circular path on the floor. Although such a standardized approach likely restricts individual variations in turning strategies, it also constrains adjustments for postural adaptation (e.g. regulation of step width).

This study has some limitations requiring consideration. Firstly, instead of quantifying COM we used a reference point (i.e. centre of pelvis segment) close to where COM is believed to be during standing and walking [22]. Secondly, BOS was calculated for each gait stride, as the distance between heel markers (see Fig 1a–1c), which leads to a marginal underestimation of the actual BOS (i.e. defined by the lateral edges of the feet). Thirdly, the horizontal velocity of the body was not incorporated into calculation of medio-lateral stability which could have influenced our results on turning stability. Our investigation was also limited to medio-lateral stability during walking turns in a comfortable and slow pace. It had been relevant to include turns at a fast speed as older adults might be more prone to instability while walking fast. Furthermore, while the study participants were healthy community-dwelling older adults, our findings might not be generalized to individuals with impaired mobility and health. Finally, as we did not investigate a group of young individuals, it is uncertain whether the turning strategies observed represent age-related adaptation to the internal perturbations induced by turning. Therefore, future studies should compare turning stability between individuals in different age spans.

## Conclusions

Older adults enhanced their medio-lateral stability during turning (i.e. increasing their margins of stability) compared to straight walking, which might reflect an effort to compensate for the greater postural challenges associated with turning. Surprisingly, we found that medio-lateral stability during turning was hampered (i.e. pelvis lateral displacement closer to the boundary of BOS), while walking speed was reduced. This finding could reflect an appropriate adaptive strategy due to the reduced postural perturbations during slow turning compared to their comfortable walking speed.
